# Notes and News

**Published:** 1887-03-26

**Authors:** 


### Notes and News.

COLLECTED AND COMMUNICATED.

SIR SPENCER WELLS, the distinguished physician, accompanied by his son and daughter, sailed last Saturday in the s.s. Opobo, for the Canary Islands and Madeira. Sir Spencer has undertaken the trip partly for pleasure and partly on account of his health.

MR. HENRY IRVING, Mr. J. C. Parkinson, and Sir Henry Brownrigg have just been elected members of the committee of the Hospital for Diseases of the Throat, Golden Square. The attendance at the out-patient department of this institution has now grown so large that its extension is becoming an imperative necessity.

A JUBILEE movement is now on foot in Bath, to raise £5,000 for the endowment of a Convalescent Home, which has been generously given to the city.

A HOUSE-TO-HOUSE Jubilee collection is proceeding at Hastings, one object of the fund being the freeing of the Hastings, St. Leonards, and East Sussex Hospital from debt. Among the larger recent contributors to this fund are the following:—Messrs. Beeching and Co., £80; Mr. G. H. Lake, £50; Miss A. Braham, Rev. J. W. Tottenham, and Colonel Tubbs, £40 each; Miss Sayer, £25; Rev. C. L. Vaughan, £15; and Mr. D. H. Gabb, £10.

WE are glad to note from the report of the Mold Cottage Hospital that the financial status of the institution was more satisfactory at the close of 1886 than the beginning of the year had enabled the committee to hope. The expenses of 1885 exceeded the income by £24 13*s*. 4*d*., but last year there was a favourable balance of £7 16*s*. 4*d*. This improvement is stated to be mainly due to the collections made by the operatives of the various works in the neighbourhood, and the subscriptions of their employers. A letter of grateful thanks for the care and kindness he had experienced addressed by a late patient to the committee, is published in the report, and affords satisfactory testimony to the conduct of the institution.

THE largely increasing number of applicants who attend the out-patient department of the St. John's Hospital for Diseases of the Skin, has induced the board of management to open the out-patient department every evening, instead of only three evenings (Monday, Wednesday, and Friday) as hitherto. The new system came into operation on Monday last.

THE annual general meeting of the governors, donors, and subscribers will be held at the University College Hospital, Gower Street, in the Board Room, on Thursday next, the 31st inst., at four o'clock, for the purpose of receiving the report and statement of accounts, and also of electing by ballot fourteen members of the hospital committee and two auditors.

MR. ROBERT FREWER, secretary of the Hospital Saturday Fund, states that in consequence of the recognition and support accorded by the postal authorities and the City police to the Morley House Seaside Convalescent Home for Working Men at St. Margaret's, Dover, the committee of management, consisting of representative working men, are now making such alterations to the house as will enable them to accommodate about 900 patients each year.

THE Durham County Hospital has recently been considerably enlarged, thanks to the munificence of Mr. John Eden. The new wards, and some other additions which have been made, will provide accommodation for about thirty more patients. One of the wards has been wisely devoted to children. The boys of Durham School have determined to subscribe annually £25 towards the support of one of the cots.

THE inhabitants of Dover have decided that their commemoration of the forthcoming Jubilee shall take the form of a new hospital for the town. Last Friday afternoon a very enthusiastic meeting was held, under the presidency of the Mayor, when a unanimous decision was arrived at. The new hospital is to cost about £5,000, and a very liberal contribution towards the sum named was subscribed in the room.

A HOSPITAL SECRETARY, whose institution is before long to be rebuilt, recently informed us that his committee did not intend to take advantage of the Jubilee as a lever for assisting the building fund, on the ground that "the thing was being a bit overdone." The board of management of the British Home for Incurables at Clapham Rise appear to be of a similar opinion, "having in view," writes the president, "the numberless collections that, for various objects, will be, and are being made this year in celebration of the Queen's Jubilee, we have decided not to hold our festival as usual. The board, in coming to this descision, feel certain that the general public, and especially those interested in this and other charitable undertakings, will acquit them of any want of loyalty to her Majesty in not following the rule of almost every other institution and society, and they also trust that this charity, which depends on voluntary offerings, will not suffer by so doing."

THE following vacancies are announced this week, the amount of the salary being placed in each case after the name of the institution :—

**Resident Medical Officers.**

Huddersfield Infirmary. Junior House Surgeon.—£40.
Macclesfield General Infirmary. Junior House Surgeon, doubly-qualified.—£70.
Manchester Royal Infirmary. One. Doubly-qualified.—£150.
Oldham Infirmary. House Surgeon, doubly-qualified.—£80.

**Matrons.**

Chelsea Hospital for Women.—£60.
Ryde Infirmary. Medically trained.—£50.
St. Saviour's Union Infirmary, East Dulwich.—£80.
Windsor Royal Infirmary.—£50.

**Trained Nurses.**

Bournemouth, Firs Home. One.—£20.
Grantham Hospital. One.
Hartlepool Hospital. One Night.—£25.
Lowestoft Hospital. Charge.—£25.
Oldham Infirmary. One Assistant.—£18.
Scarborough Hospital. One.—£35.

**Probationer.**

Coventry Hospital for Infectious Diseases. Lady Probationer.

---

ONLY four nurses had sent in their names up to Tuesday evening to Mr. Howard J. Collins, the secretary of the Hospitals Association, Norfolk House, Norfolk Street, London, W.C., with an intimation that they were anxious to join a National Pension Fund if the movement proves successful. This is not very encouraging as a beginning, considering there are quite fifteen thousand nurses in this country at the present time. Hospital secretaries, matrons, and other officers are eligible to participate in this pension scheme, and we hope many will signify their wishes in the matter to Mr. Collins without delay. Any criticisms or suggestions will be welcomed, and we will find room, if possible, for all contributions on the subject that may reach us. Mr. Newton H. Nixon, who wrote to propose the establishment of this Fund, will, we hope, be willing to act as one of the honorary secretaries, and we shall be glad to hear from him and from others on the subject.

---

MR. R. DENISON PEDLEY, M.R.C.S., L.D.S., Dental Surgeon to the Evelina Hospital, writes to us as follows :—"Though not a Guy's man, allow me to offer a word of tribute to the memory of Dr. Robert E. Carrington, by whose death a career has been closed which gave promise of exceptional brilliancy. Dr. Carrington was well known to many as a loving son to his widowed mother, a devoted brother and friend, unassuming and self-denying to a fault. Ever ready to help in time of need, he was a true friend of the poor. His charity was of the noblest type, for of himself he gave. In the conscientious devotion to many duties, his mental powers had long outstripped his physical strength. While battling bravely on, with but indifferent health, his body became a fertile ground for the ripening of the acute disease which—alas! too soon—has robbed us of a noble life, leaving his loved ones bowed down with sorrow, and almost broken-hearted." We can speak of Dr. Carrington as a fellow-student and colleague. Eminent as a physician of great promise, his industry, genius, and knowledge made him respected and esteemed by patients and colleagues alike. Poor Guy's has been most unfortunate of late years, as death has been all too busy amongst the medical staff, and Dr. Carrington's loss is one which will not readily be supplied.

---

MR. W. H. HUGHES, the secretary of the Cancer Hospital, Brompton, sends us the following further anecdote concerning hospital patients possessed of private means :—"With reference to the paragraph in THE HOSPITAL, on page 418, in which it is stated that a sum of about £300 was found some years ago in the clothing of a patient who had died in this hospital, I may inform you that only last year a patient came here from New Zealand, and remained here for about three months, when he died. Upon examining his clothes and boxes, we found gold and bankers' drafts to the amount of upwards of £1,400. Needless to say that the property was in due course handed over to the solicitors acting for the relatives of the deceased."

---

THE Cottage Hospital at Bourton-on-the-Water, in Gloucestershire, was the third village hospital established in England, being commenced in 1861 by Mr. John Moore, M.R.C.S., who is still one of its medical officers, and to whose energy and skill the highest praise is due. During the quarter of a century it has been at work, more than 1,000 in-patients and nearly 5,000 out-patients have received attention and advice at this institution. The new buildings, which have been in use since 1879, contain four wards which are capable of holding eight, or at need ten beds, in all. The income in 1886 amounted to £276 16*s.* 3*d.*, and the expenditure to £218 8*s.* 3*d.*, leaving a favourable balance of £58 8*s.* to the credit of the current account. The congregational collections from the various churches and chapels of the neighbourhood reached a total of £75 14*s.* 7*d.*, of which the largest individual sum was £8 6*s.* from Sherborne and Windrush parish church. The present flourishing condition of this hospital is largely due to the energy and devotion of the honorary secretary, Dr. Coles, who is ever watchful for its patients and zealous for its efficiency.

---

THE Dental Hospital of London, which was established nineteen years ago for the gratuitous relief of the poor suffering from diseases of the teeth, etc., has so increased its excellent work that its present premises in Leicester Square, large as they are, require to be still further extended. The operations performed last year reached no less than 43,745, or an excess of 4,560 on the number of the previous twelve months. Plans for increased accommodation are now in course of preparation. The income of the institution, thanks to the energy of Mr. J. Francis Pink, the secretary, is very well sustained, amounting in the aggregate last year to £1,712 17*s.* 2*d.*, against £1,654 in 1885. The post of hon. sec., which Mr. G. A. Ibbetson has so long held, has been abolished upon the resignation of that gentleman. Mr. Ibbetson remains associated with the hospital as one of its vice-presidents.

THE following sums have been recently received by the various Medical Charities, the name of the donor or the source from which they were obtained being given in each instance after the name of the Institution :—

**Donations.**

Dental Hospital of London, Leicester Square. Mr. W. Sibley, F.R.C.S., £10 10*s*. Mr. F. J. Bennett, £10 10*s*. Sir Ashley Gibbing, £10 10*s*.

Chelsea Hospital for Women. Miss Snelgrove, £10 10*s*. Mrs. Frank Wood, £10 10*s*. Mrs. W. C. Watson, £21. Anonymous, £21.

Royal Hospital for Children and Women, Waterloo Bridge Road. Marchioness of Westminster, £10 (for Special Fund for freeing the hospital from debt).

Queen Charlotte's Lying-in Hospital, Marylebone Road. Mr. F. H. Burrows, £21.

Richmond Hospital. Miss E. K. Brumley, £10 10*s*.

King's College Hospital. Anonymous Gift, per Messrs. Haggard, Hale, and Pixley, £1,000 stock. The Duke of Bedford, £100.

City of London Lying-in Hospital, City Road. The Company of Leathersellers, £10 10*s*. Mr. F. Fitch, £10 10*s*. Mr. H. W. Whitbread, £10 10*s*.

---

AT the twenty-fourth annual meeting of the officers of the New England Hospital for Women and Children, Boston, under the presidency of Mrs. Freeman Clarke, the secretary, Mrs. Ednah D. Cheney, presented a report which contained an interesting discussion on the relation between the community and the hospital. We regret that the *Boston Daily Advertiser*, to which we are indebted for the intelligence, does not give details of Mrs. Cheney's essay, which would doubtless be of much interest to many in this country who are connected with hospitals. Dr. Pagelsen, the resident physician, reported that 379 patients had undergone treatment during the year, of whom 195 were discharged well, 46 convalescent, 90 improved, 3 incurable, 3 unchanged, 5 admitted for diagnosis only; total discharged, 352. Free treatment was given to 179, and complete or part payment received from others. The deaths for the year were ten.

---

A CURIOUS story, says the *Mansfield Reporter*, attaches to the new out-patients' wing of the Nottingham Children's Hospital, which was recently formally opened by the Duchess of St. Albans. Of the £500 in hand to meet the cost, the bulk was left by an old newsvendor, whom most visitors to Nottingham will remember standing or sitting on the pavement in the Great Market Place under the Exchange clock. Something like eighteen months ago he disappeared from his post, and on the police effecting an entrance into the squalid den where he lived alone, in one of the yards between Long Row and Parliament Street, the old man was found dead. In the house were found scrip, notes, etc., to the value of several hundred pounds, and an informal will bequeathing it to the Children's Hospital. Under all the circumstances the old newsvendor's hoard would have gone to the Crown, but an influential friend of the Hospital bestirred himself in its behalf; it was pointed out that, however informal the will might be, there could be no doubt as to the intentions of the testator, and the noble work which the institution was doing and its needs were ably pleaded in the proper quarter, with the result that the money has been handed over to the hospital and the new wing built, although a further sum of £500 is wanted to complete it.

THE fifth entertainment to the in-patients of St. John's Hospital for Diseases of the Skin was given on the 5th instant, under the direction of Mrs. St. Vincent Mercier (the wife of the secretary), and was a great success, the patients and visitors showing their pleasure by enthusiastic applause. Mr. Dudley Wilson sang with much power "Non è ver" and "Queen of my heart"; Miss Amy Hill sang "Le nom de Marie," and afterwards Dr. Arne's "When daisies pied," to which her brother, Mr. Samuel Hill, added a clarionet *obbligato*. This song was encored. Mr. Hill afterwards played Beethoven's "Adelaide." Mr. L. H. Skinner scored a great success with his powerful recital of Rae Brown's poem, "My Lady's Leap," and, in response to an encore, caused much laughter by a recital of his various experiences as a listener to readings of "The Charge of the Light Brigade." He afterwards sang "The Horticultural Wife"; Mr Chas. Burton sang "I am Waiting," and "Close to the Threshold." Mrs. Alfred Gilbert pleasingly sang two songs, "Where you will" and "Rendez moi"; Mrs. Mercier united with Mr. Jno. Taylor (who acted as accompanist) in playing a duet for harp and piano, "Three Airs," by Mozart. The programme concluded with two recitations by Mr. C. W. Dixon. The next entertainment is to be given on 2nd April, by Dr. Dow.

---

HOSPITAL alms-boxes, whether they are fixed outside the institutions themselves, at railway stations, or elsewhere, are not, as a rule, very largely patronised by the public. We happen to know that at one institution at the West End, an out-door collection-box, in a street of exceptional gentility, yielded 4*s*. 4½*d*. as the people's contribution for six months. In by-gone days the donation-boxes outside the Royal Free Hospital in the Gray's Inn Road used to reveal some valuable finds in the shape of bank-notes and gold. We are pleased to see that some of the old luck has returned. The other day, when the boxes at the gates of this hospital were opened, there were found one ten-pound note, two for five pounds, £4 10*s*. in gold, together with a good sum in silver and bronze.

---

THE Royal Hospital for Diseases of the Chest, City Road, which was founded as far back as 1814, by the Duke of Kent, her Majesty's father, has now assumed a position of very great usefulness among the poor of the crowded districts of Clerkenwell, Shoreditch, St. Luke's, and Islington. In 1863 the old hospital was entirely rebuilt, but the new premises had to be enlarged in 1877. During the past year a new wing, providing seventy additional beds, was opened by Princess Beatrice. All this extension necessarily involves increased expenditure. The present work of the hospital requires an income of about £5,000 a year, and when all the new beds are full, the expenditure, it is estimated, will reach £7,000. The reliable income of the Hospital is only about £2,000, while the donations and collections last year brought the receipts up to £3,100, not including legacies. The need of more annual subscribers to this tried and old-established hospital is therefore very pressing. The festival dinner of the institution is to be held next May, when a special effort will be made to extinguish the building debt.

